# The complete *alk* sequences of *Rhodococcus erythropolis* from Lake Baikal

**DOI:** 10.1186/2193-1801-3-621

**Published:** 2014-10-21

**Authors:** Alexander Likhoshvay, Anna Lomakina, Mihail Grachev

**Affiliations:** Limnological Institute of the Siberian Branch of the Russian Academy of Sciences Ulan-Batorskaya 3, Irkutsk, 664033 Russia

**Keywords:** Lake Baikal, Natural oil seep, Alkane hydroxylases, *Rhodococcus erythropolis*

## Abstract

**Background:**

Rhodococci are bacteria able to degrade a wide range of hydrocarbons, including the alkanes present in crude oil, due to *alk* genes in their genomes.

**Findings:**

Genome sequencing of DNA from *Rhodococcus erythropolis* strain 4 (obtained from a deep-water bitumen mound) revealed four *alk* genes, and the predicted amino acid sequences coded by these genes were highly conserved, having sections up to 11 amino acid residues.

**Conclusions:**

Obtained four genes from *Rhodococcus erythropolis* were similar to corresponding genes from other bacteria collected from other environments, including marine sources. This indicated a large-scale horizontal *alk* gene transfer between bacteria from different subgenera.

## Background

Alkanes may constitute up to 88% of the volume present in natural oil, and due to their high toxicity can serve as a convenient source of energy only for oil-degrading microorganisms (van Beilen et al. 2003).

To overcome this obstacle, bacteria have learned to synthesize specific enzymes for extracting energy from *n*-alkanes. The initial step in *n*-alkane oxidation is catalyzed by a monooxygenase complex composed of an alkane hydroxylase (*alkB*), rubredoxin reductase (*alkT*) and rubredoxin (*alkG*, an electron carrier), which are known to play an important role in oil bioremediation (Kloos et al. 2006; van Beilen et al. 2003; van van Beilen and Funhoff 2007). Biodegradation starts with cleavage of the C-H bond, catalysed by the oxygenase-group enzyme alkane hydroxylase, which inserts an atom of oxygen from O_2_ into the hydrocarbon molecule. Prior to this catalysis, the enzyme must be activated by NADH (nicotinamide adenine dinucleotide) which transfers a pair of electrons from FAD (flavin adenine dinucleotide) to rubredoxin. One pair of electrons is transferred to alkane hydroxylase leading to the formation of primary or secondary alcohols [R-CH_3_ + O_2_ + NAH(P)H + H^+^ → R-CH_2_OH + NAD(P)^+^ +H_2_O], which are further converted to dicarboxylic acids (van Beilen et al. 2003).

This enzyme system was originally discovered in *Pseudomonas putida* and further research found that the genes encoding for alkane-degrading enzymes (*alk*-genes, rubredoxin, rubredoxin reductase) are located on a plasmid or chromosomes (van Beilen et al. 2001). Bacteria from different genera, including *Rhodococcus*, possess similar enzyme systems and *alk*-genes (Whyte et al. 1998; Whyte et al. 2002). Members of the genus *Rhodococcus* seem to play significant role in bioremediation of oil spills (Whyte et al. 2002) and are recognized as ideal candidates for the biodegradation of hydrocarbons due to their ability to degrade a wide range of organic compounds (Beard and Page 1998), their hydrophobic cell surface and the production of biosurfactants as well as their ubiquity and robustness in the environment (Larkin et al. 1998; Warhurst and Fewson 1994).

Lake Baikal, the deepest (1637 m) and oldest (25 mln y) lacustrine reservoir on Earth, is located in the middle of Eurasia. During a 2008 exploration using Mir submersibles, natural oil seepages surrounded with “bitumen mounds” were discovered on the lake bottom (Khlystov et al. 2009). These structures are stable, inhabited by living creatures and persist even if the source of oil is depleted. One of these bitumen mounds (No. 8) contained 148 mg/g of aliphatic C_22_-C_34_*n*-alkanes (primarily C_25_) where several strains of bacteria were isolated and later identified as *Rhodococcus erythropolis* (Likhoshvay et al. 2013) by means of 16S rRNA analysis.

This study aimed to identify *alk* genes in the genome of one of these isolates (strain 4) by nucleotide and complete genome sequence analysis. Strain 4, identified as *R. erythropolis*, has four *alk*-genes which differed from each other, but were similar to corresponding genes in bacteria from other habitats.

## Materials and methods

### Bacterial strain

*R. erythropolis* strain 4 (Acc. No HQ702471), isolated from bitumen mound 8 at the natural oil seep near Cape of Gorevoi Utes (10 km offshore, depth 900 m, Central Baikal) (Likhoshvay et al. 2013).

### DNA extraction and sequencing analysis

DNA was extracted by the method of Sambrook et al. (1989) with minor modifications - enzymatic lysis followed by phenol-chloroform extraction.

Complete genome sequencing of DNA was carried out according to the manual/protocol provided with the Illumina GAIIx (India). Number of readings equalled to approximately 10 Mbp. Reassembling of individual nucleotide sequences by Velvet_1.1.02 resulted to 3897 contigs with an average length of 1.8 Kbp and a total length of 6.9 Mbp. The nucleotide sequences of the *alkB* genes were translated into amino acid sequences by the Expasy Translate Tool (http://web.expasy.org/tools/translate/) and uploaded to the NCBI data base with the following accession numbers: alkane hydroxylase 1 (KF498365), alkane hydroxylase 2 (KF498366), alkane hydroxylase 3 (KF498367), alkane hydroxylase 4 (KF498368).

Homology between the four sequences was estimated by BLASTX (http://blast.st-va.ncbi.nlm.nih.gov/Blast.cgi) where the nucleotide sequences and inferred amino acid sequences were aligned with homologous sequences retrieved from GenBank using the CLUSTAL W software. A phylogenetic tree for the genes was constructed by the neighbor-joining method (Saitou and Nei 1987) using the MEGA4 program (Tamura et al. 2007). The relative synonymous codon usage (RSCU) was computed for the *alkB* genes and correspondence analysis was performed using CODONW software.

## Results

**Figure 1:**
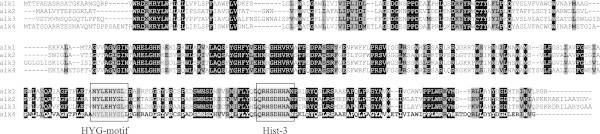
**Alignment of the amino acid sequences corresponding to the** ***Rhodococcus erythrypolis*** **strain 4.** One conserved His boxes (Hist-3) and the additional HYG motif are boxed and shaded gray. Amino acid residues, conserved in all alkane hydroxylases, are in black colour. The degree of conservation at each position was created using Clustal X.

**Figure 2:**
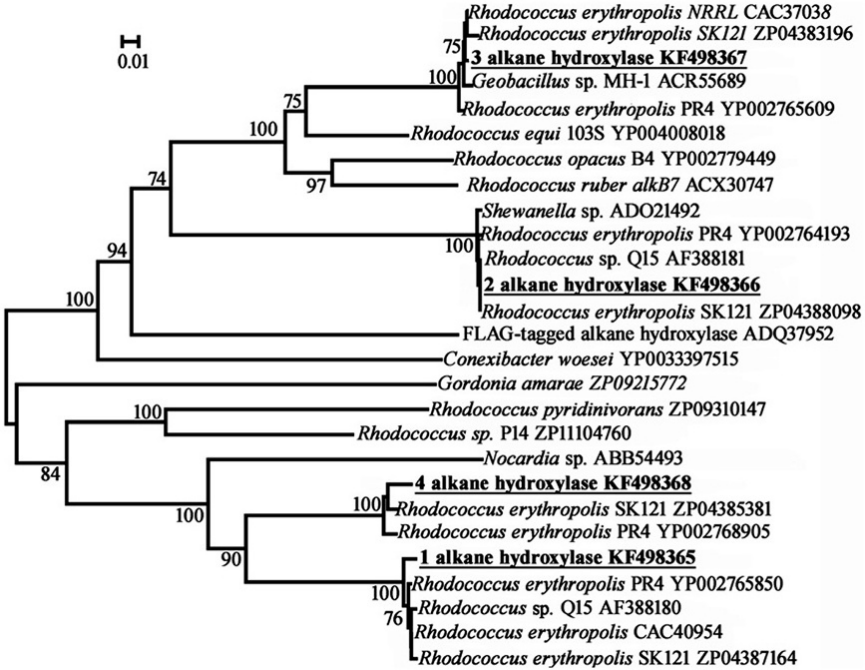
**Phylogenetic tree based on amino acid sequence alignment for** ***alkB*** **genes from** ***R. erythropolis*** **strain 4 (in bold).** Scale bar, 0.01 substitutions per amino acid site. Numerals indicate the statistical reliability of the branching order as determined by bootstrap analysis of 100 alternative trees. Values exceeding 70% were considered significant.

Examination of the complete genome DNA sequence of *R. erythropolis* strain 4 (isolated from the inner part of a bitumen mound) revealed four *alk* genes: *alk*1 was 386, *alk*2 was 389, *alk*3 was 408 and *alk*4 was 400 amino acids in length. All four sequences for these alkane hydroxylases had homologous, synonymous and significant substitutions (Figure 1). The highly conserved sequences, typical of amino acid sequences (HE[L/M]xHK, EHxxGHH, LQRH[S/A]DHHA) from alkane-degrading bacteria (van Beilen et al. 2005), were found to have the following sequences - HELGHK, EHNxGHH and LQRHSDHHA. According to data obtained by phylogenetic analysis (Figure 2), the alkane hydroxylases were located on four different branches of the tree with corresponding sequences from other rhodococci.

**Figure 3:**
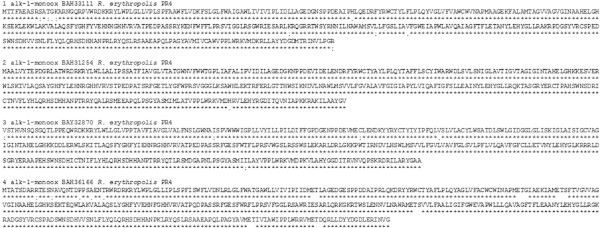
**Amino acid sequence alignment of the four new alkane hydroxylases from** ***R. erythropolis*** **strain 4 from Lake Baikal with another strain already in the literature (** ***R. erythropolis*** **PR4).** Conserved regions (*), synonymous substitutions (:), significant substitutions (∙) and unspecified substitutions (_).

Complete amino acid sequences obtained from *R. erythropolis* strain 4 were compared to the recently published genome (NCBI) of *R. erythropolis* PR4 (Sekine et al. 2006) (Figure 3) and found to be nearly identical: alkane hydroxylase 1–9 substitutions including 6 synonymous (97.6% identity); alkane hydroxylase 2–1 nonsynonymous substitution (99.7% identity); alkane hydroxylase 3–5 substitutions including 4 synonymous (98.7% identity); alkane hydroxylase 4–2 synonymous, 3 nonsynonymous substitutions and 5 inserts of 2–3 amino acids (94.2% identity).

Comparative analysis of a wide range of homologous eubacterial sequences (NCBI) revealed that the genome of *R. erythropolis* strain 4 contained highly conserved areas. In the sequence of the fourth alkane hydroxylase (BAH36166) – EHNFGHH – polar histidine (H) with basic properties was substituted for a nonpolar hydrophobic phenylalanine (F). The same substitutions were only found in corresponding sequences of *R. erythropolis* SK121 (ZP04385381) and *R. erythropolis* PR4 (YP002768905).

## Discussion

*AlkB* genes in the genomes of Gram-positive and Gram-negative alkane-degrading bacteria are usually present as several individual copies (van Beilen et al. 2003). In particular, *R. erythropolis* NRRL B-16531 and *R. erythropolis* Q15 possess four *alkB* homologues and suggests these bacteria tend to have several *alkB*-genes encoding for alkane hydroxylase (Whyte et al. 2002). In the genome of the *R. erythropolis* strain 4, 4 nucleotide sequences for (oxygenase group) alkane hydroxylases were identified. A 5th *alkB* gene has also been identified which encoded for rubredoxin reductase, but did not cluster the other 4 and will be discussed in later articles.

The alkane hydroxylase amino acid sequence homologies between *R. erythropolis* strain 4, *R. erythropolis* SK121 and *R. erythropolis* PR4 are remarkable for the following reasons: strain SK121 (Hamamura et al. 2008) was isolated from oil contaminated soil and tends to utilise aromatic hydrocarbons. Strain PR4 was isolated at a depth of 1 km from the Pacific Ocean and is unable to utilise arenes, but does use *n*-alkanes with chain length of C_8_-C_20_ as the sole energy source (Sekine et al. 2006). The *R. erythropolis* strain 4 was isolated from the inner part of bitumen mound, located on the bottom of Lake Baikal and tends to utilise *n*-alkanes with a broader chain length (C_12_-C_29_). This adaptation could be explained by the composition of the bitumen mound 8, which included *n*-alkanes with chain lengths of C_22_-C_34_ (Likhoshvay et al. 2013). However, as a final product of alkanes biodegradation serve fatty acids with chain length of C_16_-C_18_. These substances could further be degraded during phospholipid synthesis (Alvarez 2010).

Homologue sequence analysis (NCBI) of the 4 amino acid sequences from *R. erythropolis* strain 4 revealed that the 4 alkane hydroxylases were highly divergent, however each enzyme was similar to the corresponding homologue from *Rhodococcus*. The absence of other bacterial genera in the analyses suggested this was an enzyme system specific to rhodococci, based on the differences in alkane hydroxylases.

All alkane-degrading bacteria have alkane hydroxylases containing the following three sequences: (numbering from *Pseudomonas putida* GPo1): H_138_E[L/M]xHK_143_, E_167_HxxGHH_173_ and L_309_QRH[S/A]DHHA_317_. According to van Beilen et al. (2005), a histidine in the second and third sequences may affect enzyme activity. Furthermore, the histidine residues in these conserved sequences bind two atoms of Fe(II) in the alkane hydroxylase (Whyte et al. 1999; van Beilen et al. 2005). The longest sequence, L_309_QRH[S/A]DHHA_317_, was present in the alkane hydroxylase sequences of most hydrocarbon-oxidising bacteria, including *R. opacus* B4, which was initially isolated from oil contaminated soil and metabolized a wide range of arenes and aliphatics. The genes coding for these enzymes were located in (at least) six replicons: a large linear chromosome of 7,913,450 bp, two linear plasmids - pROB01 (558,192 bp) and pROB02 (244,997 bp), and three circular ones – pKNR (111,160 bp), pKNR01 (4,367 bp) pKNR02 (2,773 bp). Originally isolated at a depth of 1 km in Pacific Ocean, *R. erythrypolis* PR4 had a circular chromosome of 6,516,310 bp, a separate linear plasmid - pREL1: 271,577 bp and two circular plasmids – pREC1 - 104,014 bp and pREC2 - 3,637 bp. The first two code for most of the genes responsible for alkane metabolism. Obviously, plasmid *alk* genes could be transferred between bacteria by horizontal gene transfer (Turova et al. 2008). Hence, bacteria of the *Geobacillus* genus could obtain *alk* genes from *Rhodococcus*.

The least ones could be found everywhere and in different climatic zones and they have enormous biodegradation potential to utilise widest range of organic substrates. The structure of *alk* genes apparently has an adaptive character and encodes alkane hydroxylase. This might be necessary for *R. erythropolis* strain 4 to degrade of heavy *n*-alkanes, which are present in bitumen mound 8 at low temperture (3.5°С) and high pressure (90 atm).
